# Spontaneous Tumor Lysis Syndrome in an Undifferentiated Uterine Sarcoma: A Case Report and Review of the Literature

**DOI:** 10.1002/ccr3.9715

**Published:** 2024-12-10

**Authors:** Zahra Valizadeh, Parisa Farshchi

**Affiliations:** ^1^ Department of Internal Medicine, School of Medicine Imam Khomeini Hospital Complex, Tehran University of Medical Sciences (TUMS) Tehran Iran

**Keywords:** gynecologic cancer, solid tumor, tumor lysis syndrome, undifferentiated uterine sarcoma

## Abstract

Tumor lysis syndrome (TLS), as an oncologic emergency, may rarely occur in patients with solid organ neoplasms and without previous cancer therapy. Physicians should be highly aware of the possibility of TLS, with special attention in patients having bulky tumors, irrespective of recent treatment with cytotoxic agents for its prompt prevention and treatment.

## 1 Introduction

Massive and uncontrolled lysis of malignant cells results in the life‐threatening oncologic emergency known as tumor lysis syndrome (TLS), which was initially described in 1929 [1]. Extensive release of intracellular components into the systemic circulation causes metabolic disturbances such as hyperuricemia, hyperphosphatemia, hyperkalemia, and hypocalcemia [2] leading to potentially fatal consequences including acute kidney injury (AKI), seizure, cardiac dysrhythmia, and eventually death [3]. TLS, mostly known as a post chemotherapy complication of hematologic malignancies, is primarily seen in tumors that have a rapid cell growth or a high susceptibility to antineoplastic therapy. However, TLS is very rare among solid tumors. It is even more rare in patients without a history of cytotoxic agent therapy, which is commonly referred to as spontaneous TLS [2, 4]. However, it is crucial for healthcare providers to be aware of the risk factors and early signs of TLS to implement preventive strategies effectively and to provide prompt treatment when needed in solid tumors as well as hematologic malignancies. Treatment typically involves aggressive intravenous hydration, management of electrolyte abnormalities, and administration of uric acid‐lowering agents [5]. Here, we report an extremely rare case of uterine sarcoma that was complicated by spontaneous TLS.

## 2 Case History/Examination

A 51‐year‐old woman with a history of undifferentiated uterine sarcoma was admitted to our hospital with complaints of nausea, vomiting, oliguria, and abdominal distension. She was diagnosed with uterine sarcoma 10 months prior to admission and treated by total abdominal hysterectomy with bilateral salpingo‐oophorectomy (TAH‐BSO) followed by 10 sessions of radiotherapy and four cycles of chemotherapy, which ended 5 months before admission. On initial evaluation, the patient was conscious and alert, her blood pressure was 97/67 mmHg, pulse rate 82 beats/min, temperature 37.0°C, and oxygen saturation 95% while breathing ambient air. Her physical examination was notable for severe abdominal distention and mild tenderness all over the abdomen.

## 3 Differential Diagnosis, Investigations, and Treatment

The initial laboratory results were remarkable for hyperkalemia (6.2 meq/L), hyperuricemia (18.9 mg/dL), hyperphosphatemia (6.4 mg/dL), and AKI (creatinine 2.8 mg/dL with her creatinine at baseline of 0.6 mg/dL). The patient's laboratory results are summarized in Table 1.

**TABLE 1:** Patient's laboratory data during hospitalization.

Laboratory value	On admission	Day 7th	Day 14th	Reference range
Leukocytes (× 10^3^/μL)	6.1	7.9	4.8	4–10
Hemoglobin (g/dL)	11.2	11	9.5	12–16
Platelets (× 10^3^/μL)	335	403	215	150–450
Urea (mg/dL)	104	99	95	15–50
Creatinine (mg/dL)	2.8	3	2.3	0.7–1.4
Uric acid (mg/dL)	18.9	17.5	13.3	2.3–6.6
Calcium (mg/dL)	7.2	7	7	8.6–10.2
Phosphorous (mg/dl)	6.4	4	3.5	2.5–5
Potassium (meq/L)	6.2	5.6	3.8	3.5–5
Sodium (meq/L)	137	138	147	135–145
Magnesium (mg/dL)	1.6	2.1	2	1.6–2.6
Albumin (g/dL)	2.9	2.8	2.8	3.5–5.2

**FIGURE 1:**
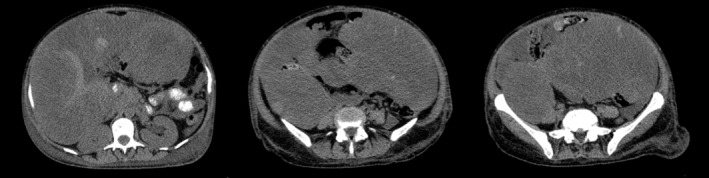
CT scan of abdomen and pelvic demonstrating multiple masses with extensive invasion to adjacent organs.

Multiple masses were observed in the abdominal and pelvic computed tomography (CT) scans (Figure 1), demonstrating extensive uterine neoplasm.

Considering the aforementioned electrolyte imbalances and a new onset of AKI along with evidence of bulky tumors, the patient fulfilled the Cairo–Bishop criteria [5] and was diagnosed with TLS.

Immediately thereafter, aggressive intravenous fluid therapy as well as oral Allopurinol were initiated. Moreover, intravenous Rasburicase was administered after confirmation of glucose‐6‐phosphate dehydrogenase (G6PD) sufficiency, and the patient planned for renal replacement therapy with hemodialysis.

## 4 Conclusion and Results (Outcome and Follow‐Up)

Despite all the mentioned treatment and several cycles of hemodialysis, the patient's condition deteriorated, and eventually she died because of multiorgan failure.

## 5 Discussion

Tumor lysis syndrome, as a serious oncological emergency, is caused by the breakdown of intracellular elements like potassium, phosphorus, and nucleic acids. It is diagnosed using the Cairo–Bishop laboratory and clinical criteria [5]. High tumor cell proliferation rate, large tumor burden, high sensitivity of malignant cells to cancer therapy, preexisting renal failure, dehydration, elevated pretreatment serum lactate dehydrogenase (LDH), and pretreatment hyperuricemia are the most important risk factors responsible for developing TLS [6].

TLS is most frequently observed in patients with hematologic malignancies, particularly high‐grade lymphomas and acute lymphoblastic leukemia following initiation of cytotoxic therapy [7]. However, TLS has been rarely described in solid organ tumors. In 2022, Alqurashi et al. conducted a systematic review reporting manifestations of TLS in 132 solid tumors. The most common solid neoplasms complicated by TLS were as follows: hepatocellular carcinoma, lung cancer, breast cancer, melanoma, colon cancer, and prostate cancer. Most of the reported cases occurred after initiation of cancer treatment and only 24% were spontaneous [7].

Among solid tumors, gynecologic malignancies, such as uterine cancers, very rarely result in TLS [3]. There are very limited published cases in the literature on uterine neoplasms‐associated TLS, with majority of cases developed TLS after the initiation of cytotoxic agents. Our literature review revealed only six cases of endometrial cancer developing spontaneous TLS [4, 8, 9, 10, 11]. Features of the reported cases are summarized in Table 2, highlighting the severity and mortality of spontaneous TLS in gynecologic malignancies despite their rarity.

**TABLE 2:** Overview of reported cases of endometrial cancers complicated with spontaneous TLS.

Patient's age (Years)	Cancer histology	Outcome	Authors/Year
58	Uterine leiomyosarcoma	Death	Alaigh et al./2017 [9]
59	Dedifferentiated endometrial adenocarcinoma	Death	Harada et al./2017 [4]
33	Endometrioid endometrial adenocarcinoma	Alive	Berger et al./2017 [8]
65	Uterine serous adenocarcinoma	Death	Berger et al./2017 [8]
59	Poorly differentiated adenocarcinoma with neuroendocrine feature	Death	Azanza et al./2020 [10]
75	Endometrial adenocarcinoma	Death	Zhao et al./2021 [11]

To the best of our knowledge, our case is the first case of undifferentiated uterine sarcoma complicated with spontaneous TLS. As in our patient, Ahmed et al. reported a case of undifferentiated endometrial stromal sarcoma with TLS in a 71‐year‐old woman, though its occurrence was associated with the administration of carboplatin and paclitaxel [12].

Although TLS in solid neoplasms, especially gynecologic cancers, remains rare, it is a life‐threatening condition with high morbidity and mortality [11], and previous studies showed a greater mortality rate for TLS in solid neoplasms compared to hematologic malignancies [13, 14]. Recent studies highlight the importance of recognizing specific characteristics and parameters related to a worse prognosis. They emphasize that metastatic disease, especially in the liver or lungs, is significantly associated with spontaneous TLS‐related death compared to no metastasis [7]. We presented an extremely rare case of spontaneous TLS in a gynecologic malignancy with no prior recent chemotherapy. Such finding highly suggests that immediate risk stratification of patients for TLS prophylaxis as well as prompt diagnosis and treatment are crucial in solid organ tumors. Despite the relatively low incidence of spontaneous TLS in solid malignancies, physicians should take its possibility into account, particularly in the case of aggressive solid tumors, irrespective of recent treatment with cytotoxic agents, to reduce morbidity and mortality. Further research is needed to better understand the pathophysiology of spontaneous TLS in solid tumors and predictors of morbidity and mortality in spontaneous TLS and to develop targeted strategies for effective prevention and management in patients with solid tumors, including those with gynecologic malignancies.

## 6 Conclusion

Gynecological malignancies can at times be complicated with spontaneous TLS with high morbidity and mortality, necessitating physicians' awareness for timely diagnosis, prevention, and treatment.

## Author Contributions

**Zahra Valizadeh:** data curation, formal analysis, investigation, writing – original draft, writing – review and editing. **Parisa Farshchi:** conceptualization, data curation, formal analysis, investigation, project administration, supervision, writing – original draft, writing – review and editing.

## Ethics Statement

The authors declare that appropriate written informed consent was obtained for the publication of this manuscript and accompanying images. This study was approved by the research and ethics committee of Tehran University of Medical Sciences.

## Consent

Written informed consent was obtained from the patient to publish this report in accordance with the journal's patient consent policy.

## Conflicts of Interest

The authors declare no conflicts of interest.

## Data Availability

The data that support the findings of this study are available from the corresponding author upon reasonable request.
